# Stability and Formation of the Li_3_PS_4_/Li, Li_3_PS_4_/Li_2_S, and Li_2_S/Li Interfaces: A Theoretical Study

**DOI:** 10.1021/acs.langmuir.3c02354

**Published:** 2023-12-11

**Authors:** Naiara Leticia Marana, Silvia Casassa, Mauro Francesco Sgroi, Lorenzo Maschio, Fabrizio Silveri, Maddalena D’Amore, Anna Maria Ferrari

**Affiliations:** † Theoretical Group of Chemistry, Chemistry Department, Torino University, 10124 Torino, Italy; ‡ Gemmate Technologies s.r.l., Buttigliera Alta, Torino, 10090 Italy; § Department of Chemistry and NIS, University of Turin, 10125, Torino, Italy; ∥ Istituto Nazionale di Ricerca Metrologica, 10135 Torino, Italy; ⊥ CNR-Nano and CNR-ITAE - National Research Council, 00185 Roma, Italy

## Abstract

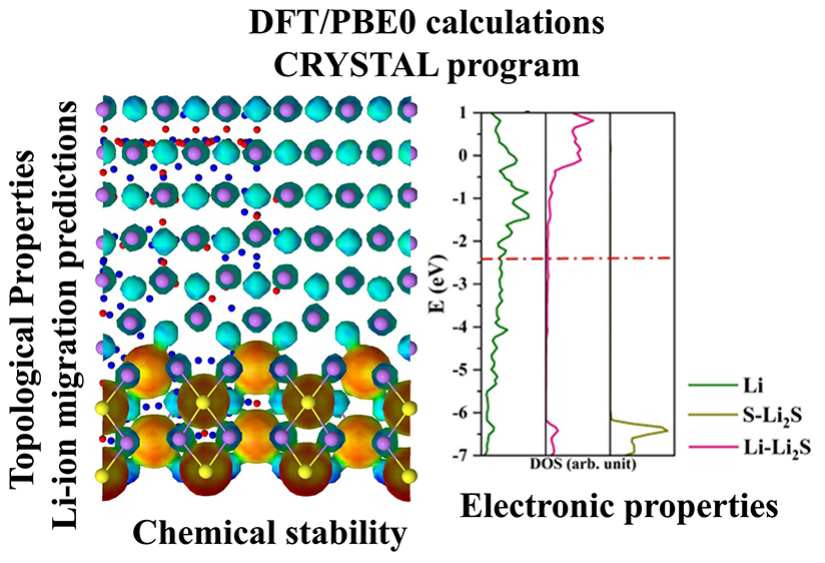

Solid electrolytes have shown superior behavior and many advantages over liquid electrolytes, including simplicity in battery design. However, some chemical and structural instability problems arise when solid electrolytes form a direct interface with the negative Li-metal electrode. In particular, it was recognized that the interface between the β-Li_3_PS_4_ crystal and lithium anode is quite unstable and tends to promote structural defects that inhibit the correct functioning of the device. As a possible way out of this problem, we propose a material, Li_2_S, as a passivating coating for the Li/β-Li_3_PS_4_ interface. We investigated the mutual affinity between Li/Li_2_S and Li_2_S/β-Li_3_PS_4_ interfaces by DFT methods and investigated the structural stability through the adhesion energy and mechanical stress. Furthermore, a topological analysis of the electron density identified preferential paths for the migration of Li ions.

## 1 Introduction

In recent years, lithium batteries have become some of the most used batteries in electronic devices. However, the increasing technological requirements continuously push toward more efficient batteries, with a long cycle life and high specific energy.^1^ In all-solid-state lithium batteries (ASSLBs),^2^ the electrolyte is composed of a semiconductor (crystalline or amorphous) solid electrolyte (SE) that, compared to traditional liquid electrolytes, has some advantages, such as superior thermal stability, lower flammability, and improved durability, but also some disadvantages, such as chemical incompatibility with electrodes, electrochemical reaction, and mechanical issues. SE enables the use of Li metal as an anode that ensures high capacity (∼3860 mAh g^–1^)^3^ to the battery but, at the same time, because of the very low reduction potential (−3.04 V vs the standard hydrogen electrode), may cause the chemical decomposition of many solid electrolytes. Therefore, a good solid electrolyte must guarantee not only a high ionic conductivity but also good electrochemical stability under a bias to avoid additional reactivity due to the presence of a voltage window and good chemical stability in order to preserve the structural integrity once coupled with highly oxidative cathodes and highly reductive anodes. Sulfide-type materials^4−6^ have shown good performance as solid electrolytes,^7^ and these materials have drawn a lot of attention due to their low grain-boundary resistance, easy processability, and good structural compatibility.

In particular, the sulfur-based electrolyte β-Li_3_PS_4_ (lithium thiophosphate - LPS) has a structure with partially occupied Li-ion sites that promotes high ionic conductivity, 3.0 × 10^–2^ S cm^–1^ at 573 K.^8^ Nevertheless, for the effective use of LPS as SE, good chemical compatibility and adherence with all the other materials and main components of the battery are required: this implies the formation of stable solid-electrolyte interfaces (SEI)^9^ and, at the same time, requires the guarantee of good Li-ion transfer across the device. In ASSLBs, the most critical issue occurs at the contact between SE and the lithium anode where, during battery cycling, unfavorable (electro)chemical reactions lead to the decomposition of the SEI, reducing the ionic conductivity and causing a dramatic deterioration of battery performance. Along with this problem, the formation of lithium dendrites can cause short circuits in the device, posing serious limits to the functionality of the overall systems and requiring considerable conceptual and technological efforts in order to make sulfur-based SEs operational.

To solve these issues, several strategies have been proposed. One of them is to use Li alloys, as an anode or electrolyte, to prevent the electrochemical reduction of solid electrolytes and the formation of Li dendrites,^10^ such as Li–Cu, Li–Mg alloys,^11^ and Li–Au.^12^ Some of the lithium alloys have a higher lithiation/delithiation potential,^13^ but the lower Li content significantly compromises the maximum cell energy density that the lithium metal anode can deliver. Another strategy is the use of thin films of a material interplayed between the solid electrolyte and the Li anode, called functional buffer layers, that preserve the advantageous properties of the interface and prevent the occurrence of any detrimental chemical reaction or modification; for this purpose, materials such as LiF^14^ and lithium sulfide (Li_2_S)^15,16^ have been suggested in the literature due to their range of electrochemical stability with respect to Li metal. The solid electrolyte can also be doped by inorganic materials forming solid solutions that will increase the adhesion with the Li-metal negative electrode; for instance, the use of LiI^17^ as a dopant not only improves the Li-metal compatibility but also the Li-ion conductivity.

Although some studies report good compatibility among Li, LPS, and Li_2_S separately, as far as we know, no one has carried out a complete characterization of the chemical stability of the two-dimensional interfaces obtained by coupling the three materials in any relevant way, regarding their static properties, i.e., the analysis of the bonding framework, surface restructuring, thermodynamic stability, chemical reactivity, and at the same time their transport properties related to possible migration paths of Li atoms across the interfaces.

The choice of the two materials to be combined with metallic lithium was made after careful analysis of the most recent literature. Regarding the LPS, we demonstrated in a previous work^18^ that the most stable structure belongs to the Pn2_1_a group (a Pnma subgroup) and presents Li atoms in the 4a Wyckoff position; this peculiar Li occupancy could modify the interface formation and play an important role in the Li-ion migration mechanism. As far as Li_2_S is concerned, (i) it has good compatibility for sulfur-based solid electrolytes,^19−21^ (ii) it is an electrochemically inactive material with a high potential barrier, (iii) and despite the low ionic conductivity at 400 K of pristine Li_2_S of ∼10^–5^ S cm^–1^,^22,23^ the increase in structural defects associated with temperature and also the method of preparation^24^ could increase in its ionic conductivity, and these properties can make Li_2_S a promising candidate as a passivating material to stabilize the SEI structure. Accordingly, we present here a theoretical study based on accurate *ab initio* calculations to propose the Li_2_S film as a possible material to be used as a passivating layer between the solid electrolyte LPS and the (110) surface of the Li metal.

The article is arranged as follows: Computational details and structural models are presented in the next section. In the second section, we discuss the main results on interface adhesion and strain as well as electronic and topological properties. In the last section, some general conclusions are drawn.

## 2 Theoretical Methodology and Models

The calculations were performed using the CRYSTAL23 package,^25^ within the density functional theory (DFT) approach, combining the hybrid PBE0 functional^26,27^ and the all-electron basis sets 6-11G,^28^ 86-311G*,^29^ and 85-211dG^30^ for Li, S, and P, respectively. The adopted computational scheme, which was already used in a previous study^18^ on the bulk and surfaces of β-Li_3_PS_4_, has been shown to provide structures and energies in good accordance with data available in the literature.

In the CRYSTAL code, the accuracy of the truncation criteria for the bielectronic integrals, Coulomb and HF exchange series, is controlled by a set of five thresholds for which the strict values of [8, 8, 8, 8, 16] were adopted. The reciprocal space was sampled according to a sublattice with shrinking factor 8, corresponding to 34 and 16 independent *k* points in the irreducible part of the Brillouin zone for Li_2_S/LPS, LPS/Li, and Li_2_S/Li.

Dealing with the case of interfaces with metallic behavior to make SCF convergence less sensitive to the position of Fermi energy and to the density of the sampling *k* grid, a Fermi–Dirac smearing procedure was adopted with a value of 0.01 hartree for the corresponding parameter (0.27 eV). The basis set superposition error (BSSE) was estimated *a posteriori* by applying the standard counterpoise method.^31^

To model surfaces and interfaces, the CRYSTAL code adopts periodic slab models with two infinite dimensions (*x* and *y*) and a finite thickness along the *z* direction. Three interfaces were considered: (1) a 4-layer slab of Li_2_S (110) grown on a 7-layer slab of Li (110) referred as the substrate (Li_2_S/Li); (2) a 4-layer slab of Li_2_S (110) grown on 8 units of a LPS (100) slab called the substrate (Li_2_S/LPS); and (3) 8 units of an LPS (100) slab grown on a 7-layer slab of Li (110) (LPS/Li). The stability of these heterostructures was estimated by considering the corresponding adhesion energy (per surface unit), *E*_adh_, computed as
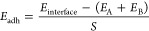
1where *E*_interface_ is the total energy of the heterostructure, *E*_A_ is the total energy of the substrate, *E*_B_ is the total energy of the overlayer, and *S* is the surface area of the interface. The substrate A has been kept fixed at the bulk lattice parameters and defines the lattice parameters of the interface, whereas the overlayer B was structurally modified in order to match the substrate: the energy cost for this deformation defines the strain energy (per surface unit), *E*_strain_, which has to be taken into account for a proper estimate of the overall stability of the composite. *E*_strain_ was computed as
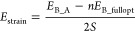
2where *E*_B*_*fullopt_ is the energy of the fully relaxed overlayer B and *E*_B*_*A_ is the energy of the overlayer B optimized at the lattice constants of the substrate A (that is at the lattice parameters defining the interface).

The choice of the surfaces involved in heterostructure formation has been guided by considerations over the surface stabilities computed as usual
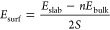
3where *E*_slab_ and *E*_bulk_ are the slab and bulk energies, respectively.

Geometry optimizations were carried out using analytical gradients with respect to atomic coordinates, and the convergence threshold for atomic forces was set to 0.0045 eV/Å; about the Li substrate, three Li bottom layers have been kept fixed at the bulk values during the geometry optimization.

Topological properties were obtained by using the TOPOND program embedded in the CRYSTAL code. The topological analysis of charge density according to the quantum theory of atoms in molecules and crystals (QTAIMAC) proposed by Bader^32^ represents an accurate tool to understand the interactions and chemical bonds in materials. This theory is based on the analysis of the charge density, ρ(**r**), and attributes a correspondence with chemical objects and concepts between the critical points, CP, of this function. In particular, (i) the minima, maxima, and points of inflection of the charge density are searched as the points where the first derivatives of ρ(**r**) vanish and then classified according to the sign of the three nonzero eigenvalues (3, *x*) of the matrix of its second derivatives (3, −3), (3, −1), (3, +1), and (3, +3). Three negative signs, (3, −3), indicate a global maximum in the charge density, and then the point corresponds to the position of an attractor, i.e., the nuclei of the system structure; the (3, −1) CP is a maximum in two directions and a minimum along the direction connecting two attractors and then represents a bond critical point (BCP) between two atoms; and finally, (3, +1) and (3, +3) are minima of the density in two and three directions, respectively, and define regions of charge depletion that usually correspond to rings (RCP) in two dimensions and cages (CCP) in three dimensions. A BCP indicates the presence of an interaction between atoms, and in this way, it was possible to follow the breaking and the formation of bonds between the surfaces. Furthermore, based on the values of some quantities calculated in the bond critical point, ρ(**r**_BCP_), it was possible to classify the chemical interaction as ionic, covalent, or belonging to the transition region between the two. The topological indicators are the electronic density itself, the Laplacian, ∇^2^ρ(**r**_BCP_), the potential energy density, *V*(**r**_BCP_), the positive definite kinetic energy density, *G*(**r**_BCP_), and the bond degree, *H*(**r**_BCP_)/ρ(**r**_BCP_), with *H*(**r**_BCP_) = V(**r**_BCP_) + G(**r**_BCP_). Finally, ring and cage critical points, RCP and CCP, respectively, were used to predict possible migration paths for the Li ion across the interfaces, based on the assumption that these regions may represent corridors along which charged species can move while undergoing a minor Coulomb repulsion.

The electron density and charge potential (electron/bohr^3^) are calculated on a regular grid of three-dimensional dots between 0.00/0.01 and −0.01/0.01 a.u., respectively. The isodensity surfaces are calculated at regular intervals of 0.02 a.u., and CUBE format files are generated. To identify the positive (red) and negative (blue) regions, the electrostatic potential is superimposed on the charge density.

## 3 Results and Discussion

### 3.1 LPS, Li_2_S, and Li Isolated Layers

In order to design a reliable model for each surface, we verified the convergence of the most significant observables, (i.e., structural parameters, surface energy, atomic charges, band gap, and Fermi level) with respect to the slab thickness. The relevant physical data obtained for bulk and surface structures of LPS, Li_2_S, and Li metal are reported in Table 1. The computed values are in good agreement with experimental data.^33,34^

**Table 1:** Cell Parameter (*a*, in Å), Band Gap (*E*_gap_, in eV), Fermi Level (*E*_F_, eV), Surface Energies (*E*_surf_, in eV/Å^2^), and Slab Thickness (in Å) for the Bulk Structures and the 8 Units of LPS, the 4-Layer Li_2_S, and the 7-Layer Li

		*a*	*b*	*E*_gap_/*E*_F_	*E*_surf_	*z*_thickness_
LPS	Pnma	12.91	8.14	4.75	-	-
LPS–surf	(100)	6.23	8.14	4.66	0.002	23.61
Li_2_S	Fm3m	4.03	4.03	5.17	-	-
Li_2_S–surf	(110)	3.96		5.58	0.037	6.13
Li	Im3m	3.46	3.46	–3.04	-	-
Li–surf	(110)	2.99	2.99	–2.98	0.034	14.67

In our previous study,^18^ we found that the (100) slab is not only the most stable surface for LPS, followed by the (010) and the (210), but is also the most interesting due to its porous-like surfaces which can facilitate the diffusion of the Li ion through its channels. The structure with 8 units of Li_3_PS_4_ and a negligible surface energy of 0.002 eV/Å^2^ is used here as the model for the LPS material.^18^

For Li_2_S and Li, we performed a preliminary study of the surface stability of the exposed surface. Stoichiometric slabs of increasing thickness were modeled by cutting the two bulks, Li_2_S and Li, along the (100), (110), and (111) directions. Based on the computed surface energy, the (111) and (110) surfaces of Li_2_S and the (100) and (110) surfaces of Li metal are the most stable, and this result is in line with other theoretical studies.^16,35,36^ In the case of Li_2_S, the four-layer slab (4L) is already a good model for the bidimensional material since the computed properties are almost at convergence with respect to the number of layers. For instance, for the (110) surface, *E*_surf_ is 0.0376 eV/Å^2^ for 4L and 0.0377 eV/Å^2^ for 14L and the band gap is 5.58 eV for 4L and 5.21 eV for 14L. Similarly, we found that the properties computed for the 5L slab of Li metal do not differ significantly with respect to those computed for the 14L slab: as an example, for the (110) surface, the difference in the surface energy is 0.001 eV/Å^2^ and the difference in the Fermi level is only 0.09 eV. As a result, in the following, the 5L (for Li/Li_2_S) and 7L (for Li/LPS) slabs will be used as models for Li surfaces.

### 3.2 Stability and Formation of the Interfaces

To obtain a reliable model of an interface, it is important to combine the two different materials in a way that leads to a good match between the substrate and the overlayer (i) by exploiting the chemical compatibility and obtaining a large adhesion energy and (ii) by avoiding a significant mismatch between the lattice parameters of the two subunits that can cause mechanical issues. The selected configurations are a necessary compromise between the feasibility of the model and the affordability of the calculations.

#### 3.2.1 LPS/Li Interface

As already discussed, the main disadvantage of using metallic Li as the anode is its high chemical reactivity with solid-state electrolytes. Therefore, to immediately address the main problem, we first studied the LPS/Li interface, trying to verify its chemical stability and structural integrity.

**Figure 1:**
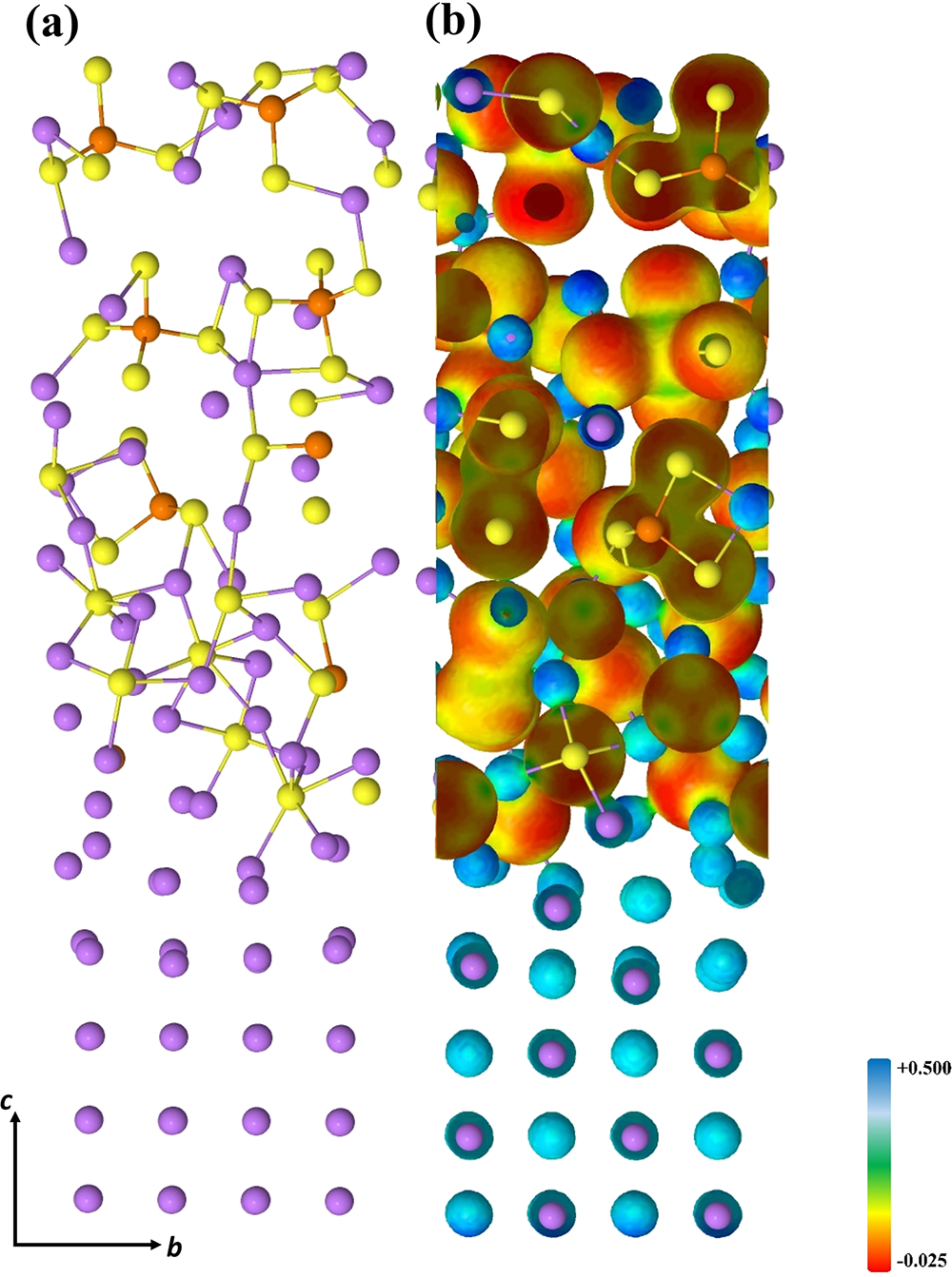
Final optimized (100)LPS/(110)Li interface: (a) side view and (b) electron charge distribution on the isodensity surface. See Figure S1 to visualize the isodensities of the free subunits.

For the LPS/Li heterostructure, 8 units of the LPS (100) slab has been combined with a (2 × 2) supercell of the 7L slab of Li (110), resulting in an interface with lattice parameters of *a*_0_ = 6.91 Å and *b*_0_ = 9.78 Å. Since the Li surface is the substrate, the three layers farthest from the interface were kept fixed during the optimization. To match the Li substrate, the LPS overlayer undergoes compressive strains of 1.28 and −6.76% along *a* and *b*, respectively, corresponding to a surface strain of *E*_strain_ = 5.67 meV Å^–2^, which is fully negligible concerning the computed adhesion energy, *E*_adh_ = −602.51 meV Å^–2^; results are reported in Table 2. In addition, the very large value of *E*_adh_ suggests a fundamental contribution to the formation of the composite due to chemical interactions which involve the breaking of existing bonds and the formation of new ones. We notice that the heterostructure is accompanied by a large reconstruction in the interface layers of the two moieties: some atoms of the LPS surface drop into the Li surface, and at the same time, some of the lithium atoms of the metal move to form bonds with sulfur. The [PS_4_] units, which initially were at the interface, undergo a major change: P–S bonds break, P atoms migrate into the metal (the inward P displacement along *z* is 4.57 Å), and Li_3_P units are formed. The final interface is composed only of Li and S atoms, which arrange to form a lattice structure resembling that of Li_2_S (see Figure 1).

**Table 2:** Energy of Adhesion (*E*_adh_), Strain (*E*_strain_), Basis Set Superposition Error (*E*_BSSE_) in meV Å^–2^, Band Gap (eV), and Atomic Charge Transfer (CT, in |e| per Cell) for the Analyzed Interfaces^a^

	*E*_adh_	*E*_strain_	*E*_BSSE_	*E*_adh_ + *E*_strain_ + *E*_BSSE_	*E*_gap_	CT
LPS/Li_2_S	–64.94	+8.14	+3.55	–52.71	4.35	0.035
Li_2_S/Li	–36.53	+0.57	+8.67	–27.30	0.00	2.548
LPS/Li	–644.61	+5.67	+42.10	–602.51	0.00	7.355

a Calculated band gaps of the (100) LPS surface and (110) Li_2_S surface are 4.58 and 5.63 eV, respectively.

The overall process corresponds to a redox reaction
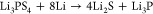
4where the metal Li is oxidized to Li^+^ and P is reduced from +5 to −3 with a total exchange of 8 electrons. Thus, the large value computed for the interface energy is indeed justified by the chemical reaction occurring at the interface, which is spontaneous and largely exothermic. We must emphasize that the spontaneity of the reaction does not lie in any instability due to mechanical stress, since the computed strain energy (Table 2) is too small to justify such a driven force, but lies exclusively in the (electro)chemical nature of the materials brought into contact. We will return to this aspect in the following section.

It is important to highlight that in our static DFT calculations we did not observe the formation of other byproducts besides Li_3_P and Li_2_S. The decomposition of several solid electrolytes in the presence of Li metal has already been well discussed in the literature,^37,38^ and regarding sulfur-based electrolytes, their decomposition into binary compounds of Li, Li_2_S, and Li_3_P is expected and leads to an increase in Li-ion transport resistance. According to Wang and coauthors,^39^ the decomposition of argyrodite (Li_6_PS_5_Cl) and the subsequent formation of Li_3_P and Li_2_S phases as an interfacial layer are crucial to the transport of Li ions, as it can increase cell resistivity. On the other hand, as highlighted by Gorai and coauthors,^40^ the appreciable electronic conductivity of Li_3_P (decomposition product) can lead to continuous reactions with Li metal and increased interface decomposition.

The decomposition of Li_3_PS_4_ into Li_2_S and Li_3_P was confirmed by several indicators. The topological analysis of the BCPs confirms the ionic character of the Li_2_S and Li_3_P bonds. Also, the new Li–S bonds, formed at the SEI, are of the ionic type, very similar to those that occur in LPS and Li_2_S, as shown in Table 3. The Hirshfeld charges, computed before and after the reaction, change as follows: from 1.551|e| to 1.013|e| for Li and from −1.112|e| to −2.061|e| for P, with an overall charge transfer from the LPS to the metal of 7.3|e| per cell, in good agreement with the charge balance required by the redox numbers in eq 4. The charge density distribution as projected onto the electrostatic potential isodensity surface, and shown in Figure 1, returns this same description showing the increase in the negative/positive charge around the S/Li atoms at the interfaces, while the charge density of atoms far from the interface remains almost unchanged (see Figure 1 and Figure S1 for a comparison with the isolated subunits).

**Table 3:** Electron Charge Density (ρ), Its Laplacian (∇^2^ρ), the |*V*|/*G* Ratio, and the Bond Degree H/ρ, All in Atomic Units, Computed at the Bond Critical Points (BCPs) in the Heterostructures at the PBE0 Level^a^

A–B	*d*_BCP__-A_	*Ρ*	∇^2^ρ	|*V*|/*G*	*H*/ρ
Li/LPS					
Li–S	0.822	0.017	0.083	0.831	0.18
Li–P	0.792	0.024	0.098	0.921	0.07
Li_LPS_–S	0.829	0.016	0.077	0.801	0.20
P_LPS_–S	0.981	0.014	–0.194	3.185	–0.62
Li/Li_2_S					
Li–S	0.785	0.023	0.113	0.858	0.16
Li–Li	1.054	0.008	0.004	1.558	–0.13
Li_Li_2_S_–S	0.783	0.023	0.115	0.877	0.13
LPS/Li_2_S					
Li_Li_2_S_–S_LPS_	0.794	0.021	0.103	0.857	0.15
Li_Li_2_S_–S_Li_2_S_	0.798	0.020	0.099	0.858	0.15
Li_LPS_–S_LPS_	0.794	0.021	0.103	0.843	0.17
P_LPS_–S_LPS_	0.983	0.134	–0.175	3.265	–0.58

a Li, Li_LPS_, and Li_Li_2_S_ refer to the atoms of Li in the Li metal negative electrode, Li in the LPS, and Li in the Li_2_S.

**Figure 2:**
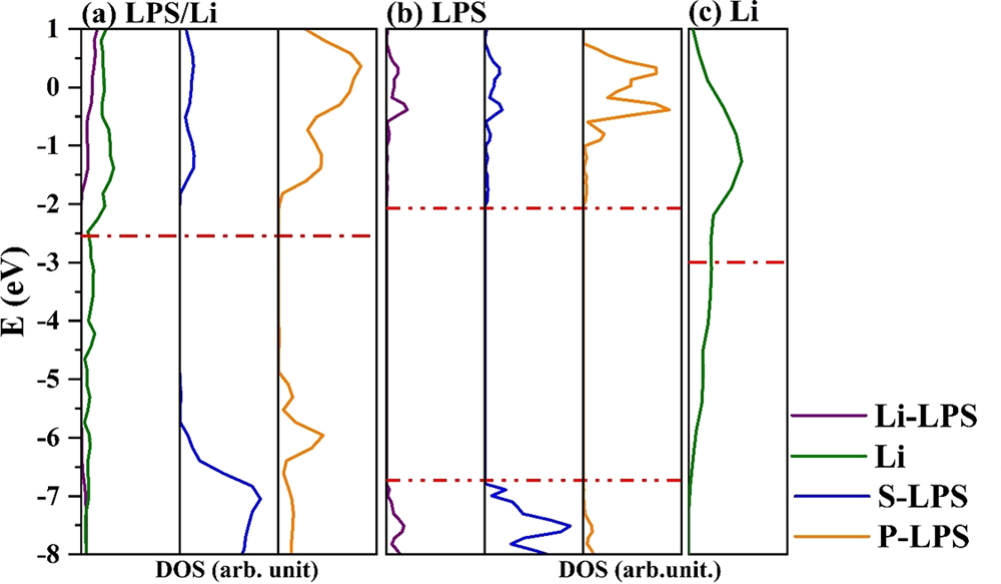
Projected density of states of the interface LPS/Li and the isolated counterparts

Then, to understand the changes induced by the interface on the electronic structure of the pristine materials, we calculated the LPS/Li projected density of states, PDOS, reported in Figure 2. The main differences are in the regions −5.5/–6.0 and −1.0/–2.5 eV, related to new electronic states due to the formation of Li–P and Li–S bonds. In addition, the upshift of the Fermi energy by almost 1 eV, with respect to the isolated Li surface (Figure 2c), is consistent with the reduction of P and its migration into the metal layer.

Our results are in agreement with some studies available in the literature on sulfide solid electrolytes. Camacho-Forero et al.^41^ simulated interfaces of different solid electrolytes (LGPS, Li_2_P_2_S_6_, Li_3_PS_4_, and Li_7_P_3_S_11_) with Li metal and verified that all of them present such deformation, although some to a lesser degree than others. For LPS, despite not having suffered a great structural deformation, the authors also observed the formation of Li_3_P and Li_2_S in the interface after 20 ps of molecular dynamics simulation. Sang and coauthors^42^ verified, experimentally, the deformation of the LPS when in contact with Li metal and the decomposition of LPS into Li_2_S. They analyzed the use of S or a LiAlO interlayer between Li and LPS, and they confirmed that both materials prevent LPS decomposition. By applying the DFT with VASP, Ji et al.^10^ analyzed the formation of Li dendrites when Li metal is in contact with LPS and forms to avoid them. They also obtained the structural deformation and Li_3_P and Li_2_S formations and suggested the use of the Li_3_N–LiF interlayer to suppress the LPS decomposition.

#### 3.2.2 LPS/Li_2_S Interface

Concerning the LPS/Li_2_S heterostructure, we proposed an interface where the Li_2_S is deposited on the LPS surface, hence acting as a coating film between LPS and a Li anode. The film has to protect the solid electrolyte from undesired redox reactions that, during the cycling of the battery, decompose the materials at the electrode interface and cause degradation of the battery performance. LPS can be considered to be the substrate, i.e., the LPS cell is the reference cell; instead, Li_2_S is the overlayer and its lattice parameters should be adapted to the LPS.

For the LPS/Li_2_S heterostructure, 8 units of the LPS (100) slab has been combined with the supercell (2 × 1) 4L Li_2_S (110) resulting in an interface with lattice parameters of *a*_0_ = 6.06 Å and *b*_0_ = 8.07 Å. To match the LPS substrate, the Li_2_S overlayer undergoes compressive strains of −8.82 and −1.23% along *a* and *b*, respectively, corresponding to a surface strain of *E*_strain_ = 8.14 meV Å^–2^ (see Table 2). The computed value for the adhesion energy is *E*_adh_ = −52.71 meV Å^–2^, an estimate that largely compensates for the strain energy.

**Figure 3:**
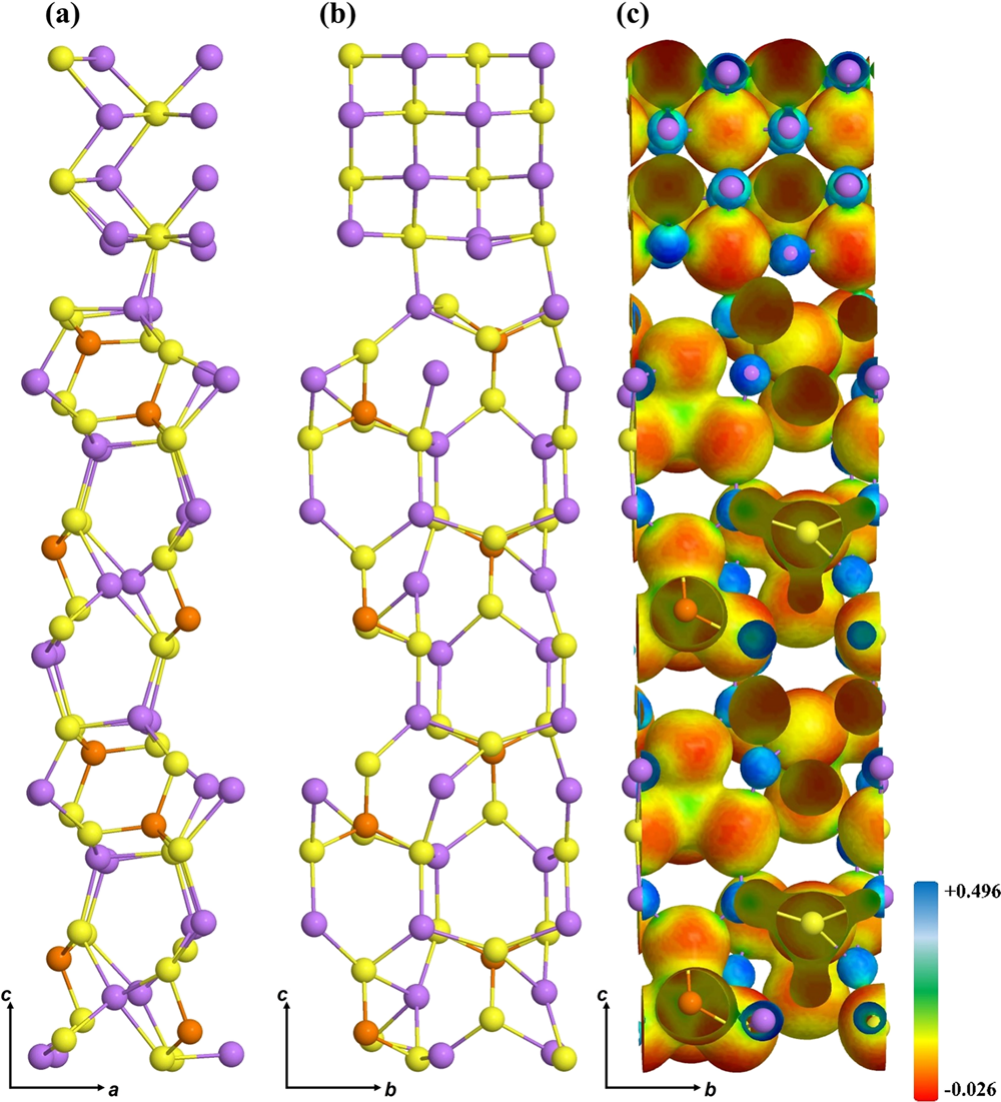
Final optimized (100)LPS/(110)Li_2_S interface: (a) side view long *ac*, (b) side view long *bc*, and (c) electron charge distribution on the isodensity surface. See Figure S1 to visualize the isodensities of the free subunits.

Looking at the interface structure, shown in Figure 3, we can appreciate a small lattice rearrangement that involves only the Li_2_S and LPS layers in close contact. The most relevant effect is a reversed distortion at the Li_2_S bottom layer, where the Li atoms are downshifted by 0.24 Å with respect to the isolate-free subunit. Similarly, the largest variation in the LPS slab is related to S belonging to the [PS_4_] cluster and the Li atom, whose distance increases by 0.23 Å. Moreover, the P–S bonds are maintained, while the Li–S bonds of the first three layers of the LPS slab are slightly elongated. At the interface, almost all Li and S ions restore their bulk coordination (8-fold for Li, 4-fold for S), with an average Li–S distance of 2.39 Å comparable to the corresponding distances in the LPS and Li_2_S counterparts (2.43 and 2.46 Å, respectively). The topological analysis confirms the formation of a Li–S bond that can be classified as ionic; see Table 3. In addition, the Li–S bond that occurs at the interface has topological properties similar to those of the LPS and Li_2_S. Finally, the charge density of the atoms not directly involved is unaffected by the formation of the interface.

These results are in agreement with other experimental and theoretical studies which evaluate the adhesion of Li_2_S to lithium–sulfur compounds.^43−45^ Wei and coauthors^20^ also analyzed theoretically the Li_2_S and LPS interface by applying the projector augmented wave method DFT/PBE, and they found good adhesion between the materials, despite the reconstruction suffered by the surfaces to form the interface bonds: the atoms belonging to first layers of the Li_2_S move toward the LPS, thus causing the detachment of some atoms from the surface of Li_2_S, forming small “agglomerates” at the interface, which was not observed in our analysis. By applying AIMD simulations, Camacho-Forero et al.^41^ studied the formation of the cathode–electrolyte interface taking into account the (111) and (001) surfaces of Li_2_S and different solid electrolytes, one of them being the LPS. They found a good, but not high, adhesion between the Li_2_S and LPS, 72.33 and 19.85 meV Å^2^, for (111) and (001) Li_2_S surfaces, respectively, and (110) of LPS. In addition, they observed that the Li_2_S surfaces showed a significant structural distortion after 20 ps of molecular dynamics simulation, which can be attributed to the high reactivity of the selected surfaces and the great strain between the Li_2_S surfaces and LPS.

**Figure 4:**
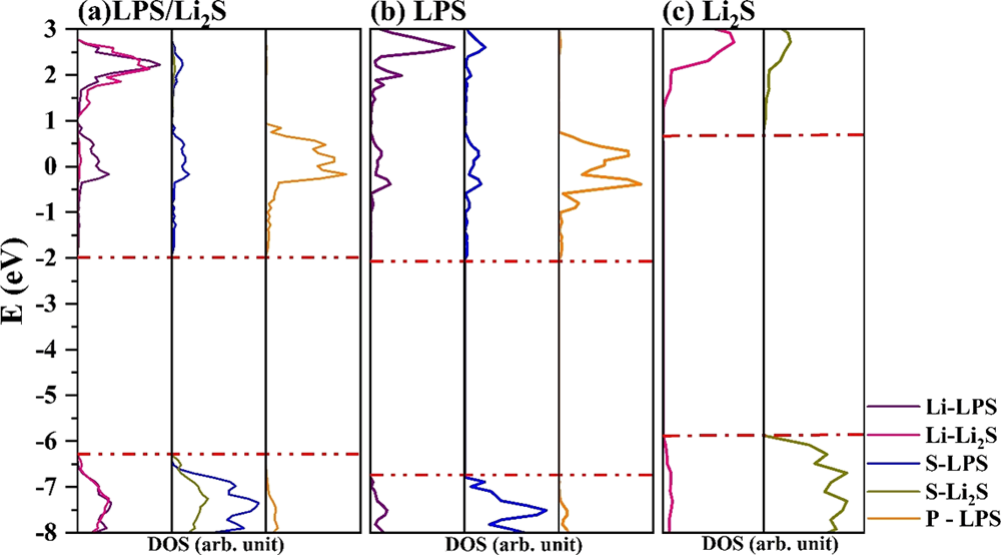
Projected density of states of the interface LPS/Li_2_S and the isolated counterparts.

The PDOS of the Li_2_S/LPS interface and their isolated counterpart are reported in Figure 4. The band gap, which was 5.17 and 4.66 eV in Li_2_S and LPS, respectively, is strongly reduced due to the band alignment and is computed to be 4.35 eV. The main overall effect is an upshift of the top of the LPS valence band, composed of Li and S states, due to the formation of new Li–S bonds at the interface.

The stability of the interface and the integrity of the surfaces involved in its formation are also evident in the analysis of the isosurfaces, Figure 3 right panel, where the only significant variation is from the atoms belonging to the interface, where the S atoms of Li_2_S become more negative and the atoms of the LPS surface become slightly positive; this finding is in line with the overall CT (see Table 2), whose value is 0.0035|e|, indicating that little (or almost no) charge is transferred from LPS to Li_2_S.

#### 3.2.3 Li_2_S/Li Interface

For the Li_2_S/Li composites, a supercell (5 × 3) 4L slab of Li_2_S (110) has been combined with the supercell (5 × 4) 5L Li (110) slab, resulting in an interface with lattice parameters *a*_0_ = 17.31 Å and *b*_0_ = 19.58 Å. With this choice for the coincidence cell, the Li_2_S mismatch to fit the Li substrate is negligible, 1.22 and 0.75% for *a* and *b* lattice parameters, respectively, yielding as expected also to negligible strain energy, +0.57 meV/Å^2^ (see Table 2), largely compensated for by the adhesion energy of the interface of *E*_adh_ = −27.30 meV Å^–2^.

**Figure 5:**
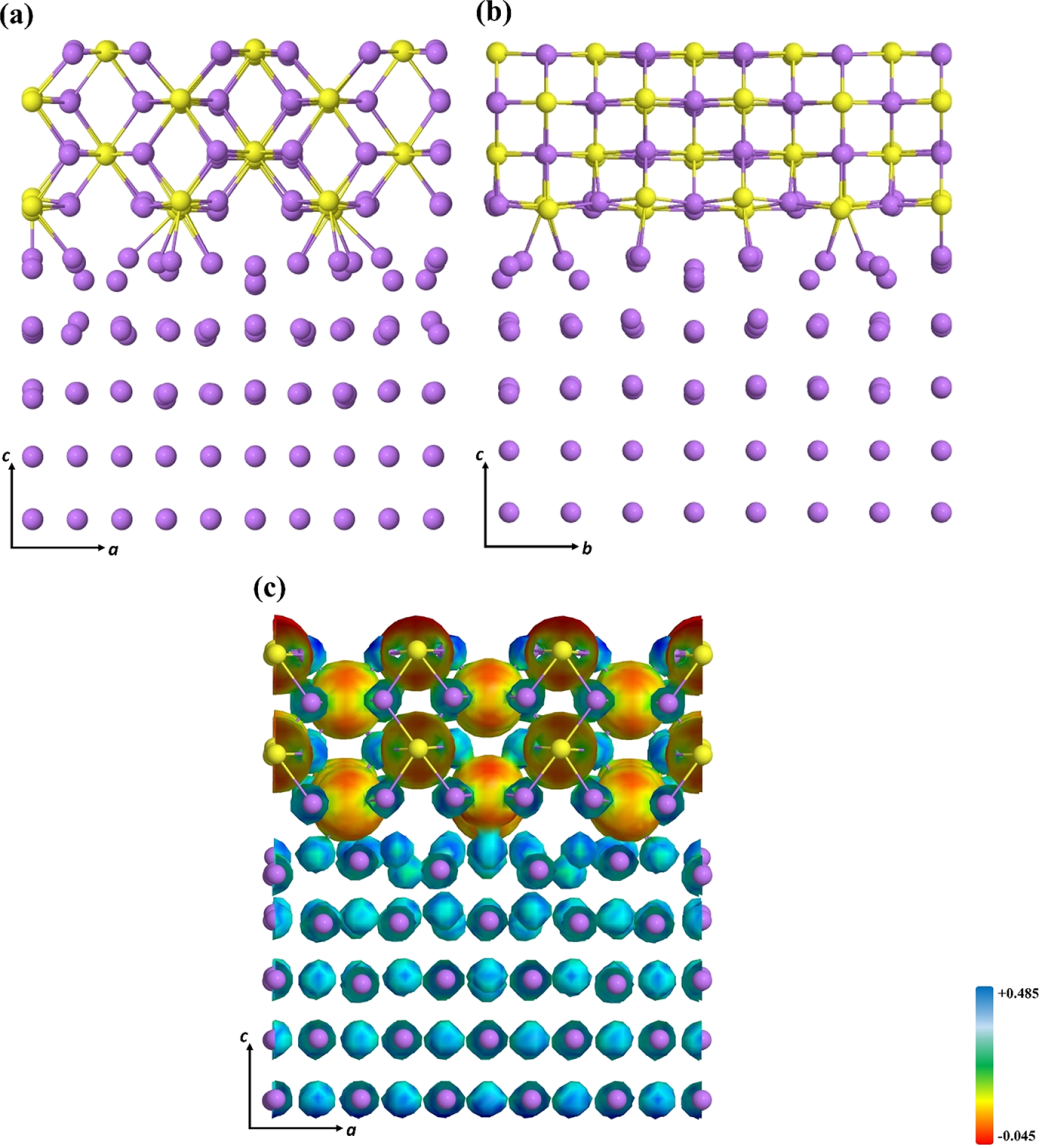
Final optimized (110)Li_2_S/(110)Li interface: (a) side view long *ac*, (b) side view long *bc*, and (c) electron charge distribution on the isodensity surface. See Figure S1 to visualize the isodensities of the free subunits.

The small mismatch and relatively good adhesion energy are confirmed by the small structural distortion of Li and Li_2_S with respect to the free counterparts. The Li-metal negative electrode involves only the two outermost layers, and the bulklike structure is recovered after the third layer (as can be seen in Figure 5). For Li_2_S, only the layer at the interface shows a small structural distortion due to a small downshift (0.56 Å) of Li atoms. New Li–S bonds are formed between the Li metal and S–Li_2_S so that all S atoms restore their 4-fold coordination. The Li–S bond distance between the Li_2_S and Li metal is 2.35 Å, close to the values computed in the Li_2_S slab (∼2.43 Å). Also, for this heterostructure, the ionic bond character of the Li–S of the interface was confirmed by the topological analysis; moreover, critical points between the Li_anode_–Li_Li_2_S_ atoms suggest the formation of a noncovalent interaction (see Table 3).

**Figure 6:**
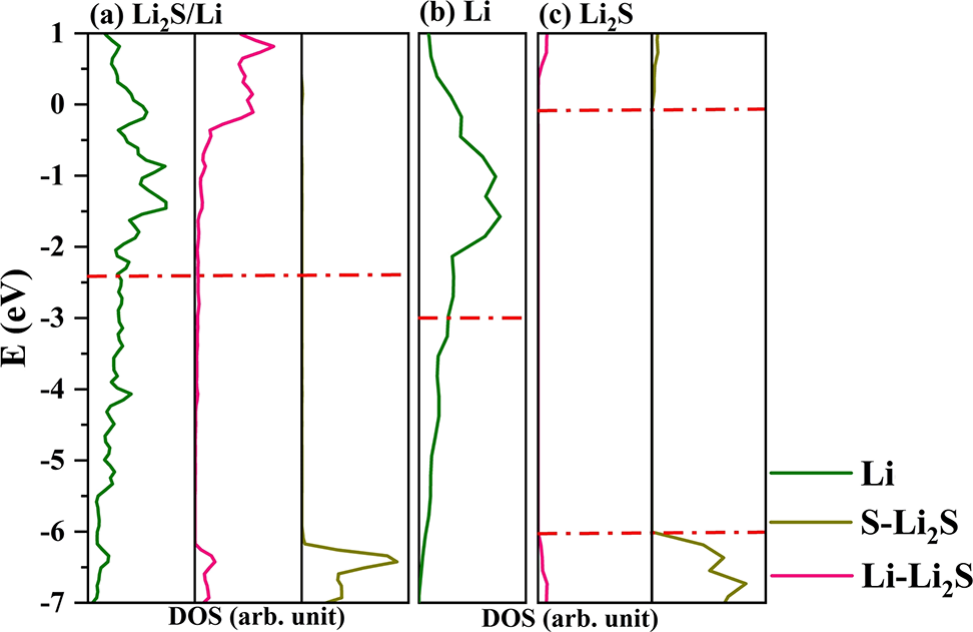
Projected density of states of the interface Li_2_S/Li and the isolated counterparts

The interface bonding is supported by the analysis of the charge density isosurfaces reported in Figure 6 and compared with those of the isolated surfaces in Figure S1. The external layer of the Li-metal negative electrode has a very well distributed charge density, and for such atoms, the calculated Hirshfield charge is zero; see Table S1. Getting closer to the interface becomes closer, the atomic population changes, and the Li atoms show values of around +0.286|e| corresponding to an accumulation of charge on the Li_2_S counterpart; Figure 6.

The electrostatic (polarization) nature of the interface can also be appreciated by the inspection of PDOS reported in Figure 6, where the main contribution to the bond formation is from the Li-metal negative electrode and S–Li_2_S states in the region of −6 to 7 eV of the valence bands together with the delocalization of charge density over the Li–L_2_S empty states, with an overall charge transfer (CT = −2.50|e| per cell) from the Li-metal negative electrode to Li_2_S, in line with a downshift of the Fermi level of −0.21 eV.

The good adhesion between Li metal and Li_2_S was also confirmed by Lai and co-workers.^16^ They investigate a form to mitigate the decomposition of the amorphous solid-electrolyte PVDF composite (sulfide–LiTFSI–poly(vinylidene difluoride)) when in contact with Li metal. From the DFT analysis, they found that the (111) surface of Li_2_S inhibits the formation of defects, in addition to presenting good wettability and low migration barrier energy, which was in agreement with their experimental data and with our results. Liu et al.^46^ also found good adhesion between Li_2_S and Li. They analyzed the Li_2_S film formation on the Li surface: the reaction is thermodynamically favorable, and the Li_2_S structures suffer less deformation when both present the same [hkl] plane.

One way to explain the stability of the Li_2_S/Li and Li_2_S/LPS surfaces and the reaction occurring at the LPS/Li interface is by analyzing the energy levels of the electrolyte’s lowest unoccupied molecular orbital (LUMO) or conduction band (CB) and the highest occupied molecular orbital (HOMO) or valence band (VB) with the chemical potential of the anode (in this case, the Fermi level).^47^ According to Goodenough and Park,^47^ if the chemical potential of the anode is above the electrolyte LUMO (CB), then the anode will reduce the electrolyte unless the anode–electrolyte reaction becomes blocked by a passivating material layer. Based on our results, the Fermi level of the (110) Li-metal negative electrode surface is ∼−2.98 eV, while the bottom of the conduction band is at −2.09 eV, corresponding to a difference of 0.90 eV, very close values that, due to the minimum existing disturbance, can cause the chemical reaction and the LPS electrolyte to be reduced. We observed that the simple contact of LPS with Li metal significantly modifies the electronic levels of LPS, and the corresponding energy levels of CB are downshifted; in particular, the contributions of the S atoms are displaced to ∼−3.13 eV, less than the Fermi level of the isolated (110) surface of Li metal (−2.98 eV). Therefore, the ∼0.90 eV difference between the conduction band and the Fermi level of the isolated counterparts is not sufficient to guarantee the chemical stability of the interface; consequently, the Li metal reduces the LPS. It should be noted that the small difference presented may be related to the level of theory used in the calculations and that a modification of the basis set or functional can modify this behavior since the difference is very small. As for the Li_2_S/Li and Li_2_S/LPS interface, the difference between the Fermi level of the Li-metal negative electrode and the bottom of the conduction band of Li_2_S and Li_3_PS_4_ is very wide, which indicates that Li_2_S cannot be reduced by Li metal and also that LPS cannot be oxidized by Li_2_S (due to the Fermi level of Li_2_S being higher than the top of the valence band of LPS). Thus, such interfaces are electrochemically stable, as confirmed by our calculations.

Therefore, in line with this, we can conclude that the Li_2_S can be applied as a coating passivating material since this material meets all the requirements: exhibits good adhesion and low strain energy with both anode and solid electrolyte materials, does not modify/or be structurally modified when the interface is formed, and does not change (significantly) the electronic properties so that ionic conductivity is maintained. However, we must emphasize that low ionic conductivity has previously been reported^39^ for pristine Li_2_S and that the formation of structural defects, such as Li^+^ vacancies and grain boundaries, is necessary to improve ionic conductivity.

### 3.3 Possible Migration Path of the Li Ion

**Figure 7:**
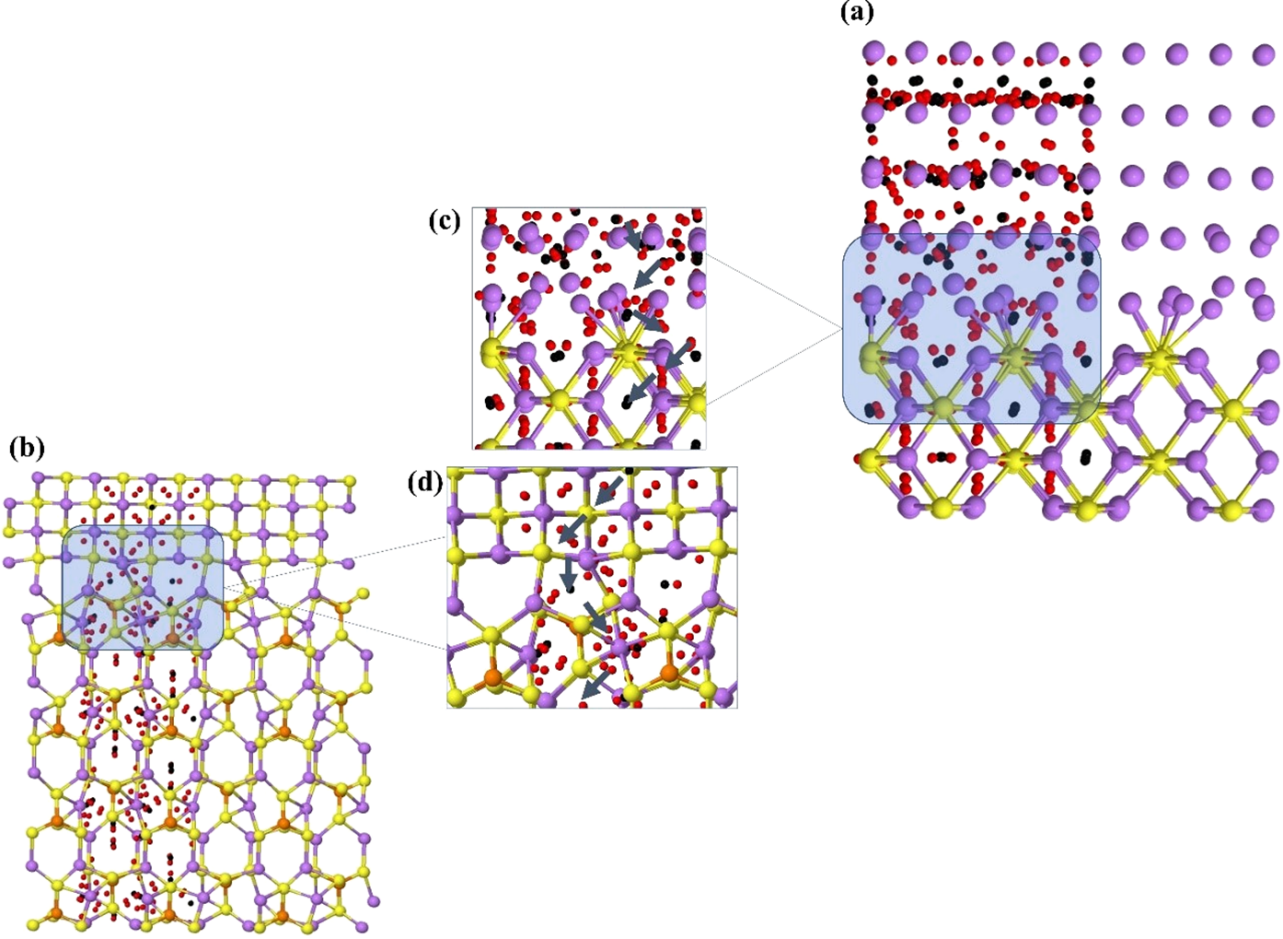
Interface structures with the (3, +1)-ring and (3, +3)-cage critical points of the charge density, in red and black, respectively, for interfaces (a) and (c) Li_2_S/Li and (b) and (d) LPS/Li_2_S. The gray arrows in (c) and (d) represent the Li migration paths along the *c* axis. Li, P, and S atoms are represented by purple, orange, and yellow, respectively.

The QTAIMC was applied as a tool to investigate the possible paths that are available for the Li ion to pass through. We analyzed the ring and cage critical points positions, RCP and CCP, respectively, of the two stable interfaces, LPS/Li_2_S and Li_2_S/Li (see Figure 7), which are represented as red and black spots. In the regions where their concentration is high, Li^+^ ions have a higher probability to diffuse through the surface due to a lower repulsion with the local chemical environment.

In a hypothetical battery model, the Li^+^ ion must leave the Li-metal negative electrode, pass through the passivating material (or coating) Li_2_S, pass through the solid electrolyte Li_3_PS_4_, and finally diffuse into the cathode. With the support of Figure 7a, we can observe that there is a large concentration of CPs along *xy* and few (and more concentrated) along *z*, on the surface of Li, see Figure 7a, which suggests that the Li ion passes from one layer to the other in very well located regions. The Li_2_S surface presents very well localized CCPs, thus, it is believed that Li-ion migration occurs through these cage-shape regions (CCP) of charge depletion. The possible migration path for this interface is shown in more detail in Figure 7c.

As for the LPS/Li_2_S interface, shown in Figure 7b, this is more ordered and less structurally deformed. The CPs are spatially very well distributed, and RCP and CCP are present in similar numbers. The R-CCPs in Li_2_S closely resemble those calculated for the Li_2_S/Li interface, so presumably the zigzag path followed by the Li ion to reach the solid electrolyte is the same. Once the ion arrives at the interface, it can drop inside a large cage with few repulsions, and then it continues passing through the Li_3_PS_4_ in a zigzag path, through regions with the highest concentration of CPs, in particular, of the CCP type, since they are structurally more spacious and provide less electrostatic repulsion.

Despite the good predictions given by this methodology,^48^ this is a preliminary analysis of the Li-ion migration, which is useful to predict the path that will be analyzed using appropriate methodologies, such as nudged elastic band (NEB) or molecular dynamic simulations, as well as the possibility to evaluate how the structural defects can modify the Li-ion migration, not only on the individual structures but also in the interface. This study is already being carried out by our group.

## 4 Conclusions

Density functional theory was applied to investigate the interface formed between the (110) surface of the Li-metal negative electrode and the (100) surface of the solid electrolyte β-Li_3_PS_4_. As discussed in some previous works, when these two materials are put in contact, many structural defects are formed and a Li_2_S structure forms in the first layers of the interface. In parallel, P atoms from the LPS surface migrate toward the Li metal and form Li_3_P and Li_2_S. Therefore, this interface becomes unsuitable for direct application in Li-ion batteries, requiring the use of a passivating material to inhibit this chemical reaction between Li and LPS. In this context, we propose the Li_2_S surface (110) as a passivating coating. Based on our calculations, we demonstrated that Li_2_S presents good adhesion energy with both Li and LPS surfaces, it is capable of creating stable interfaces, and its ionic conductivity is still preserved. It is worth noting that other factors must be considered for the long-term stability of the battery interface, such as the effect of prolonged electrochemical and mechanical bias, but our analysis of the chemical stability of the LPS/Li and Li_2_S/LPS interfaces has shown negative adhesion energies and low strain energies, suggesting that these interfaces are formed spontaneously. An in-depth analysis at the atomic level, through the topological analysis of the electron density, highlighted the presence of strong ionic bonds between Li and S, supporting the hypothesis of a mechanism of the formation of the interface driven by chemical reactions. Finally, the critical points related to regions of space where the charge is depleted showed that the Li_2_S/Li and Li_2_S/LPS interfaces have channels along which Li ions can migrate, experiencing minimal Coulomb repulsions. In our opinion, it is possible to conclude that the use of the surface (110) of Li_2_S as a passivating material could lead to a longer life for solid-state lithium batteries without affecting their performance.

## Supporting Information Available

Additional details about the pristine surfaces with their charge distribution on the isodensity surface based on the electronic charge density and the electrostatic potential (Figure S1) and the average Hirshfeld charges of the surfaces and interfaces (Table S1) calculated with CRYSTAL23 program (PDF)
