# Supplementary material for: Stability and Formation of the Li3PS4/Li, Li3PS4/Li2S, and Li2S/Li Interfaces: A Theoretical Study
Source: Langmuir. 2023 Dec 11;39(51):18797–806. doi: 10.1021/acs.langmuir.3c02354 (PMC10753886; doi:10.1021/acs.langmuir.3c02354)
Supplement: Supplementary file 1 — la3c02354_si_001.pdf [file la3c02354_si_001.pdf]

Stability and formation of the Li_3PS_4/Li, Li_3PS_4/Li_2S, and Li_2S/Li interfaces: a theoretical study

Naiara Leticia Marana[1], Silvia Casassa[1], Mauro Francesco Sgroi[1], Lorenzo Maschio[1,*], Fabrizio Silveri[2], Maddalena D'Amore[1] and Anna Maria Ferrari[1,*]

[1]Theoretical Group of Chemistry, Chemistry Department, Torino University, 10124 Torino, Italy

[2]Gemmate Technologies s.r.l. Buttigliera Alta, Italy

Supporting Information

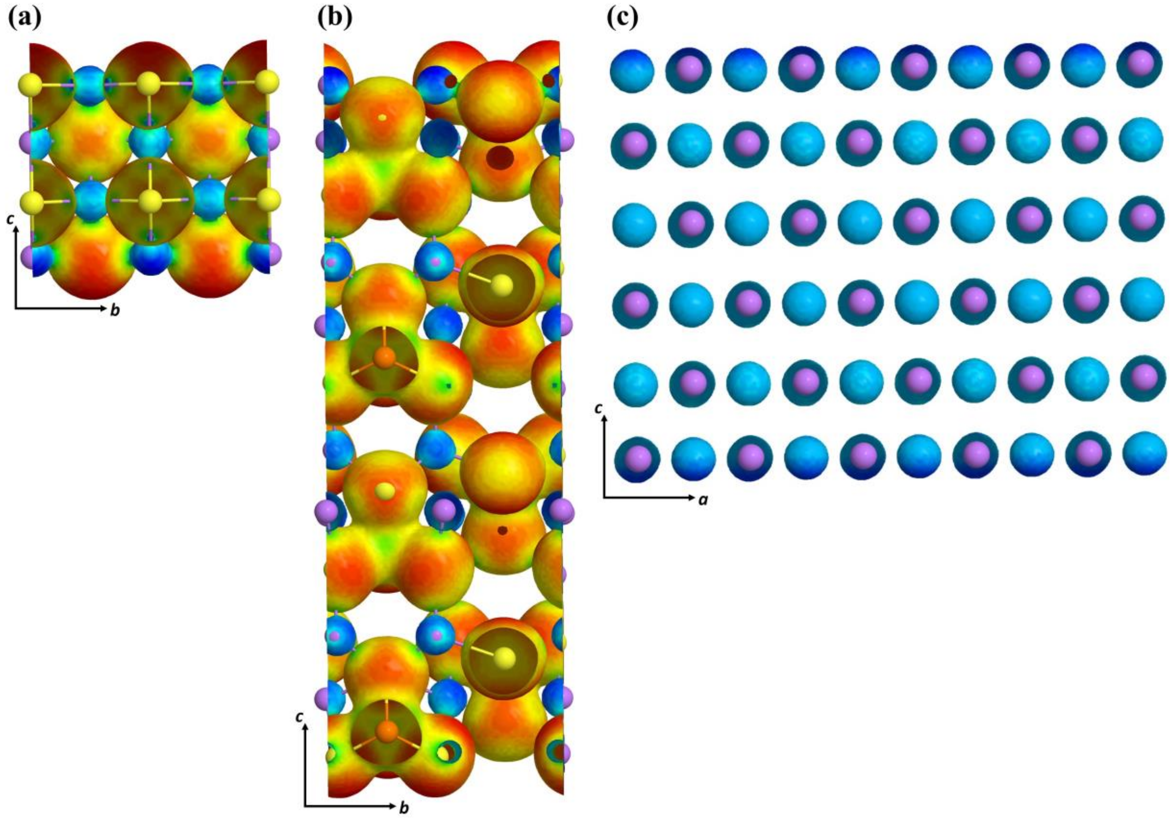

Figure S1: Optimized (a) (110) Li_2S, (b) (100) Li_3PS_4, and (c) (110) Li-metal surfaces and their charge distribution on the isodensity surface based on the electronic charge density and the electrostatic potential. The regions of blue and red color represent positive and negative charges.

Table S1: Average Hirshfeld charges (in |e| per cell) of the internal and interfacial (in) atoms. The * represents the atoms involved in the Li_3P generated during the reaction between LPS and Li-metal.

	**Li**	**S**	**P**	**$Li_{(in)}$**	**$Li_{(in)*}$**	**$S_{(in)}$**	**$P_{(in)}$**	**$P_{(in)*}$**
**Li_3PS_4 (LPS/Li_2S)**	+1.008	-1.112	+1.513	+1.015	-	-1.087	+1.553	-
**Li_2S (LPS/Li_2S)**	+1.026	-2.011	-	+1.031	-	-2.129	-	-
**Li_2S (Li_2S/Li)**	+1.018	-1.912	-	+0.637	-	-1.624	-	-
**Li (Li_2S/Li)**	0.00	-	-	+0.286	-	-	-	-
**Li_3PS_4 (LPS/Li)**	+0.989	-1.128	+1.519	+0.984	-	-1.949	-2.061	-0.459
**Li (LPS/Li)**	0.00	-	-	+1.013	+0.423			
